# PTPRT Regulates High-Fat Diet-Induced Obesity and Insulin Resistance

**DOI:** 10.1371/journal.pone.0100783

**Published:** 2014-06-20

**Authors:** Xiujing Feng, Anthony Scott, Yong Wang, Lan Wang, Yiqing Zhao, Stephanie Doerner, Masanobu Satake, Colleen M. Croniger, Zhenghe Wang

**Affiliations:** 1 Department of Genetics and Genome Sciences, Case Western Reserve University, Cleveland, Ohio, United States of America; 2 Case Comprehensive Cancer Center, Case Western Reserve University, Cleveland, Ohio, United States of America; 3 Department of Nutrition, Case Western Reserve University, Cleveland, Ohio, United States of America; 4 Department of Molecular Immunology, Institute of Development, Aging and Cancer, Tohoku University, Sendai, Japan; 5 Genomic Medicine Institute, Cleveland Clinic Foundation, Cleveland, Ohio, United States of America; University of Minnesota-Twin Cities, United States of America

## Abstract

Obesity is a risk factor for many human diseases. However, the underlying molecular causes of obesity are not well understood. Here, we report that protein tyrosine phosphatase receptor T (PTPRT) knockout mice are resistant to high-fat diet-induced obesity. Those mice avoid many deleterious side effects of high-fat diet-induced obesity, displaying improved peripheral insulin sensitivity, lower blood glucose and insulin levels. Compared to wild type littermates, PTPRT knockout mice show reduced food intake. Consistently, STAT3 phosphorylation is up-regulated in the hypothalamus of PTPRT knockout mice. These studies implicate PTPRT-modulated STAT3 signaling in the regulation of high-fat diet-induced obesity.

## Introduction

Numerous studies have shown the deleterious effects of obesity on health, increasing all-cause mortality [1] and predisposing individuals to cardiovascular disease, diabetes and cancer [2]. Diet plays a crucial role in obesity, specifically those high in fats and sugar that increase body fat [3], [4]. Adipocytes, which increase in size and number during obesity, can dramatically influence a variety of metabolic processes by disturbing normal homeostatic signals [5]. Chief among these disturbances is insulin resistance, leading to hyperglycemia and diabetes [6]–[9].

Energy imbalance – essentially a combination of increased food intake with decreased energy expenditure – causes obesity [3], [10]. Circulating hormones, such as insulin and leptin, are readouts of the body’s energy state and act at the hypothalamus to affect food intake [3], [11]–[15]. Ideally, energy intake is equal to energy expenditure, leading to weight homeostasis. However, if not enough energy is released proportional to calories consumed, the excess energy is stored as lipid in adipocytes and weight gain ensues [13]. For example, dietary fat consumption affects both sides of the energy imbalance equation. Since it releases less satiety signals in comparison to protein and carbohydrate, it leads to increased food intake [10]. Conversely, since fats are an efficient form of energy and because they are stored instead of used as an energy source after feeding, dietary lipids also contribute to decreased energy expenditure [10], [13]. Therefore, from both biochemical and physiologic perspectives of energy homeostasis, an excess of food intake over what is expended leads to weight gain.

Protein tyrosine phosphatases (PTPs) modulate signaling pathways that regulate a variety of metabolic processes through de-phosphorylating tyrosine residues on proteins [16]. Increasing evidence suggests that PTPs play a crucial role in obesity and metabolic disease [16]. It has long been known that PTP1B is implicated in obesity, insulin resistance and type-2 diabetes mellitus by regulating insulin signaling [17]. A recent study showed that TCPTP is also involved in obesity through modulating leptin signaling [18]. TCPTP dephosphorylates STAT3 at the tyrosine 705 (Y705) residue. STAT3 Y705 phosphorylation is a key mediator of leptin signaling in the hypothalamus [19], [20]. Leptin-STAT3 signaling suppresses the drive for food intake by increasing the expression of anorectic neuropeptides and repress those favoring orexigenic responses [11], [17], [18], [21], [22].

Because we previously showed that STAT3 is a substrate of protein tyrosine phosphatase receptor T (PTPRT) [23], we investigate here whether PTPRT regulates food intake and obesity in mice.

## Results

### *Ptprt* ^−/−^ Mice are Resistant to High-fat Diet-induced Obesity

**Figure 1:**
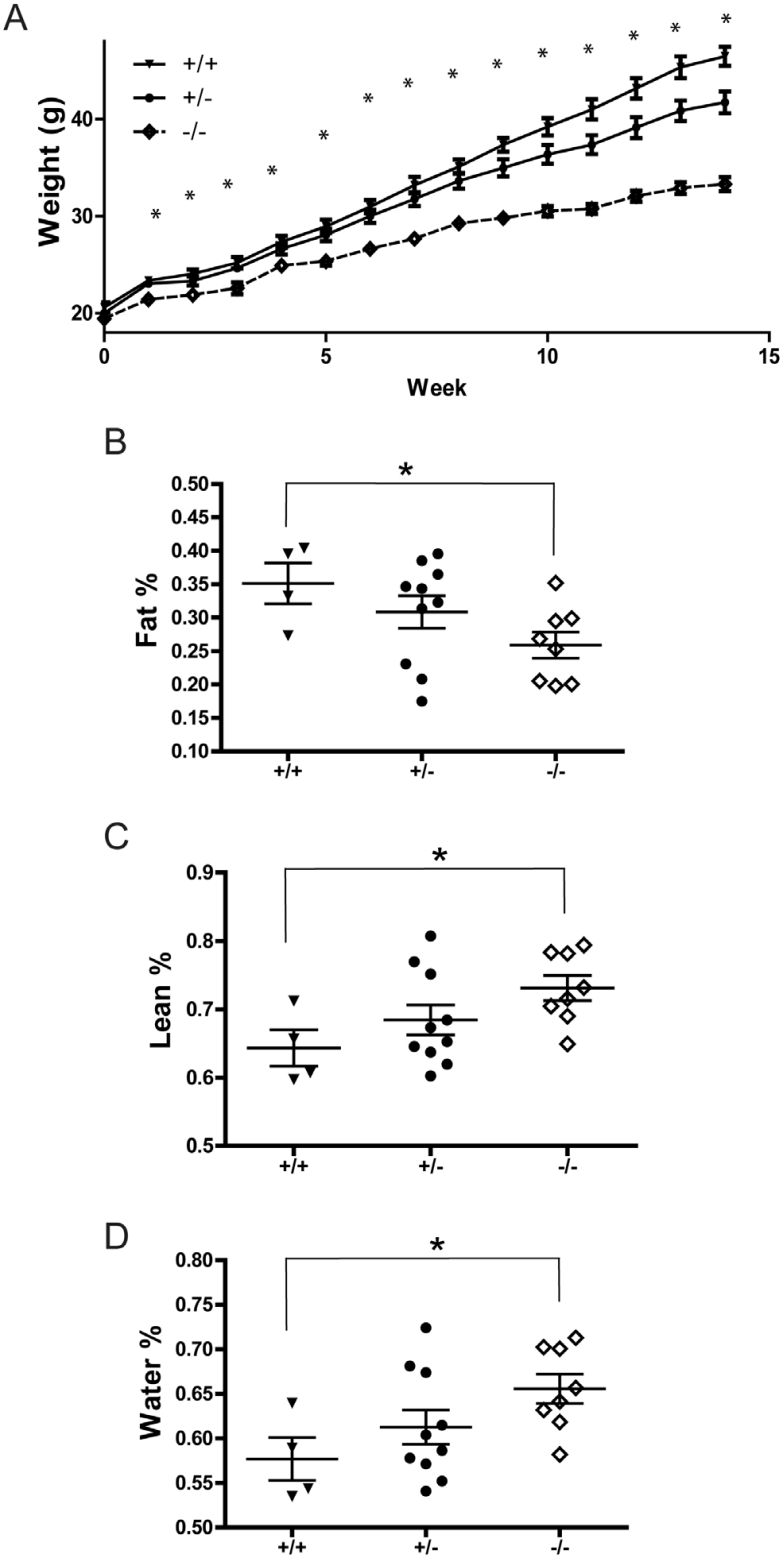
PTPRT KO mice are resistant to high-fat diet-induced body composition changes. A) Five week-old male mice of *Ptprt* ^+/+^ (n = 8), *Ptprt* ^+/−^ (n = 14) and *Ptprt* ^−/−^ (n = 11) genotypes were fed with high-fat diet for 14 weeks. Body weight of the three genotypes was assessed weekly. (*p<0.05; t-test comparing *Ptprt* ^+/+^ and *Ptprt* ^−/−^ genotypes). B–D) Body composition was analyzed by quantitative magnetic resonance after 14 weeks on a high-fat diet (Fat % – B, Lean % – C, Water % – D; *p<0.05; t-test comparing *Ptprt* ^+/+^ and *Ptprt* ^−/−^ genotypes; t-test comparing *Ptprt* ^+/−^ and *Ptprt* ^−/−^ genotypes was not significant).

As described previously [24], we bred the *Ptprt* knockout allele into the C57BL/6 strain for over 15 generations. When mouse body weights were measured from 8-week-old to 36-week-old mice, we observed that the body weight of *Ptprt* ^−/−^ mice were slightly and consistently lower than those of *Ptprt* ^+/+^ littermates on chow diet (Figure S1). However, these mice were not obese. It is well documented that high-fat diet induces obesity and insulin resistance in C57BL/6 male mice [25]. To interrogate if PTPRT plays a role in obesity, five-week-old male *Ptprt* ^+/+^, *Ptprt* ^+/−^ and *Ptprt* ^−/−^ mice were fed with a high-fat diet for 14 weeks. Although *Ptprt* ^−/−^ mice were largely indistinguishable from their *Ptprt* ^+/+^ and *Ptprt* ^+/−^ littermates in terms of body weight on a normal diet at a baseline of five weeks (Figure 1A, Time 0 weeks), the body weights of *Ptprt* ^−/−^ mice are significantly lower than those of *Ptprt* ^+/+^ littermates through the course of the high-fat diet (Figure 1A). Consistent with previous reports [25], the *Ptprt* ^+/+^ male mice were obese at the end of 14 weeks (average body weight = 46.5 g). In contrast, the *Ptprt* ^−/−^ male mice remained lean (average body weight = 33.3 g) after being fed with a high-fat diet for 14 weeks, suggesting that knocking out of *Ptprt* renders male mice resistant to high-fat diet-induced obesity.

### *Ptprt* ^−/−^ Mice have Less Body Fat by Percentage than Wild Type Littermates

Obesity and its associated co-morbidities are caused by excess amounts of body fat. Therefore, we set out to determine body composition of the high-fat diet fed mice using quantitative magnetic resonance to ensure that the difference in weight gain can be attributed to increased obesity [26]. As expected, the percentages of body fat of *Ptprt^+/+^* and *Ptprt^+/−^* mice were significantly higher than that of *Ptprt* ^−/−^ mice (Figure 1B). Consistently, the lean body mass and water in *Ptprt^+/+^* mice were lower than the *Ptprt* ^−/−^ mice (Figure 1 C and D). Since the body weight and body fat percentage of *Ptprt* ^+/−^ mice were not significantly different from those of *Ptprt* ^+/+^ mice (Figure 1), we focused on *Ptprt* ^+/+^ versus *Ptprt* ^−/−^ mice for in depth analyses in this study.

### *Ptprt* ^−/−^ Reduces Food Intake

**Figure 2:**
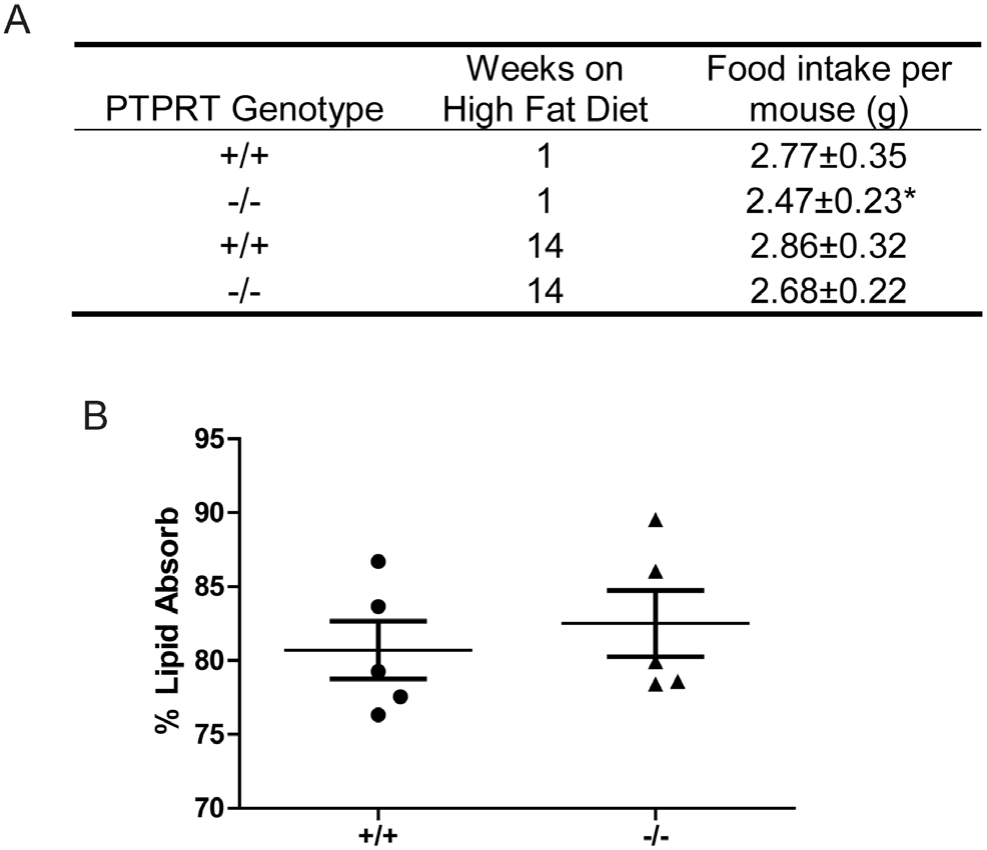
PTPRT KO mice eat less but do not absorb dietary fats differently. A) Average daily food intake of *Ptprt* ^+/+^ (n = 8), and *Ptprt* ^−/−^ (n = 12) mice was assessed at beginning and end of the high-fat diet (*p<0.05; t-test). B) Dietary lipid absorption was assessed using fecal samples of *Ptprt* ^+/+^ and *Ptprt* ^−/−^ mice after being fed a butter oil and sucrose behenate diet.

Next, we set out to interrogate the variety of mechanisms by which *Ptprt* ^−/−^ mice are resistant to high-fat diet-induced obesity. Given that food intake is one of the major factors that impact body weight, we measured food intake of *Ptprt* ^+/+^ and *Ptprt* ^−/−^ mice both at the beginning and at the end of the high-fat diet. For a ten-day period, food intake was measured daily and average values were calculated. As shown in Figure 2A, *Ptprt* ^−/−^ mice ate significantly less than their *Ptprt* ^+/+^ counterparts at the beginning of the high-fat diet period. However, at the end of the high-fat diet period, *Ptprt* ^−/−^ mice again trended toward lower food intake, but the difference did not reach statistical significance (p = 0.17).

To determine the reason behind the increased food intake in *Ptprt* ^+/+^ versus *Ptprt* ^−/−^ mice, we inferred that neurohormonal signals may play a role. Chief among these signals are leptin and neuropeptide Y, serving in an anorectic or orexigenic fashion, respectively. While plasma levels of leptin did not show significant difference between *Ptprt* ^+/+^ versus *Ptprt* ^−/−^ mice (Figure S2), *Ptprt* ^+/+^ mice had a significantly higher plasma level of neuropeptide Y versus *Ptprt* ^−/−^ mice at the beginning of the high-fat diet period (Figure S3A). This difference disappeared by the end of the high-fat diet period (Figure S3B). The plasma NPY appears to correlate with the food intake patterns at the beginning and end of the high-fat diet period.

Since dietary lipid absorption also impacts body weight and because PTPRT is expressed in the small intestine and colon [24], we set out to assess the lipid absorption capacity of *Ptprt* ^+/+^ and *Ptprt* ^−/−^ mice using non-invasive fecal analysis [27]. However, these two cohorts did not show any difference in absorbing dietary fats (Figure 2B). Taken together, our data suggest that the reduced body weight of *Ptprt* ^−/−^ mice may be due to less food consumption.

### *Ptprt* ^−/−^ Mice have Reduced Energy Expenditure than Wild Type Mice

**Figure 3:**
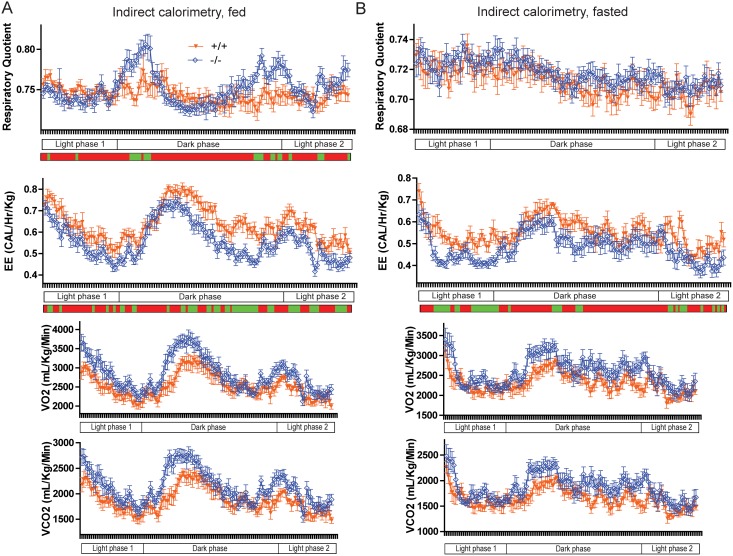
PTPRT KO mice utilize more glucose and expend less energy than wild type mice. Energy expenditure (Heat Released and Respiratory Quotient) was assessed through indirect calorimetry for mice in fed (A) and fasted (B) state through 24 hours (green segments: *p*<0.05; t-test). *Ptprt* ^+/+^ (n = 8); *Ptprt* ^−/−^ (n = 11).

Since reduced energy expenditure and differences in nutrient utilization could also contribute to obesity, we assessed the energy expenditure and respiratory quotient of *Ptprt* ^+/+^ and *Ptprt* ^−/−^ mice via indirect calorimetry in both the fed (Figure 3A) and fasted (Figure 3B) state. According to the energy expenditure values, *Ptprt* ^−/−^ mice had reduced energy expenditure than *Ptprt* ^+/+^ mice in both the fed and fasted state (Figure 3, second row). However, reduced energy expenditure in *Ptprt* ^−/−^ mice does not explain the weight difference between *Ptprt* ^+/+^ and *Ptprt* ^−/−^ mice. The respiratory quotient of *Ptprt* ^+/+^ and *Ptprt* ^−/−^ mice was indistinguishable in the fasted state (Figure 3B, top panel). In the fed state, *Ptprt* ^−/−^ mice had a higher respiratory quotient than *Ptprt* ^+/+^ mice during the dark phase (Figure 3A, top panel), indicating they preferentially use glucose, but this difference was not sustained through the whole 24-hour period of testing. Taken as a whole, indirect calorimetry demonstrates that the metabolic phenotype of *Ptprt* ^−/−^ mice does not explain their reduced body weight versus their *Ptprt* ^+/+^ littermates.

### *Ptprt* ^−/−^ Mice Resist High-fat Diet-induced Hyperglycemia and Insulin Resistance

**Figure 4:**
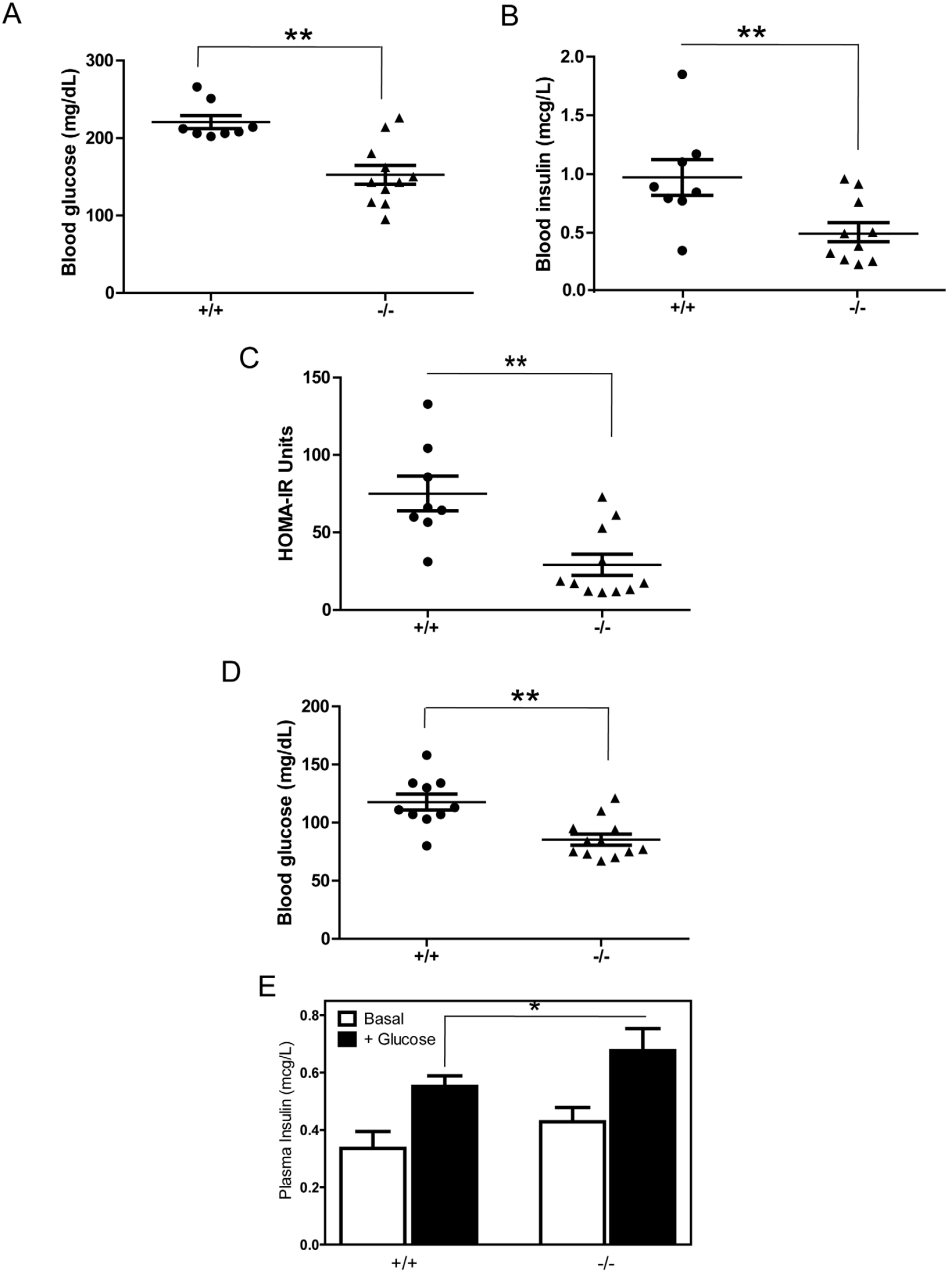
PTPRT KO mice have less insulin resistance than wild type mice after high-fat diet. A) Fasting blood glucose levels were assessed after 14 weeks on a high-fat diet (**p<0.01; t-test). B) Fasting insulin levels were assessed after 14 weeks on a high-fat diet (**p<0.01; t-test). C) HOMA-IR calculations of *Ptprt* ^+/+^ and *Ptprt* ^−/−^ mice after high-fat diet (**p<0.01; t-test). D) Fasting blood glucose levels were assessed before high-fat diet (*p<0.05). E) Glucose stimulated insulin secretion test between *Ptprt* ^+/+^ (n = 5) and *Ptprt* ^−/−^ (n = 5) mice before high-fat diet (*p<0.05; t-test).

Given that obesity often causes metabolic syndrome, such as hyperglycemia and peripheral insulin resistance, we measured fasting glucose and insulin levels in *Ptprt^+/+^* and *Ptprt* ^−/−^ littermates. At the end of the high-fat diet treatment, the average blood glucose levels in *Ptprt* ^+/+^ mice reached 220.6 mg/dL; therefore, these mice were hyperglycemic. In contrast, the blood glucose levels in *Ptprt* ^−/−^ were within normal range at 152.6 mg/dL (Figure 4A). Accordingly, *Ptprt* ^+/+^ mice also had higher insulin levels than the *Ptprt* ^−/−^ counterparts after the high-fat diet (Figure 4B). These blood glucose and blood insulin values can be used to estimate peripheral insulin resistance using the HOMA-IR model [28]. By this calculation, *Ptprt* ^−/−^ had much lower HOMA-IR values than their *Ptprt* ^+/+^ littermates (Figure 4C), indicating a prediction of insulin sensitivity versus *Ptprt* ^+/+^ mice. It is worth noting that the *Ptprt* ^+/+^ mice were neither hyperglycemic nor insulin resistant before high-fat diet treatment, although the blood glucose levels in the *Ptprt* ^+/+^ mice were slightly higher than that of *Ptprt* ^−/−^ littermates (Figure 4D). No blood insulin difference was observed before high-fat diet treatment among the *Ptprt* ^+/+^ and *Ptprt* ^−/−^ mice (data not shown). Before the high-fat diet, *Ptprt* ^−/−^ mice also secreted more insulin in response to a glucose bolus (Figure 4E).

**Figure 5:**
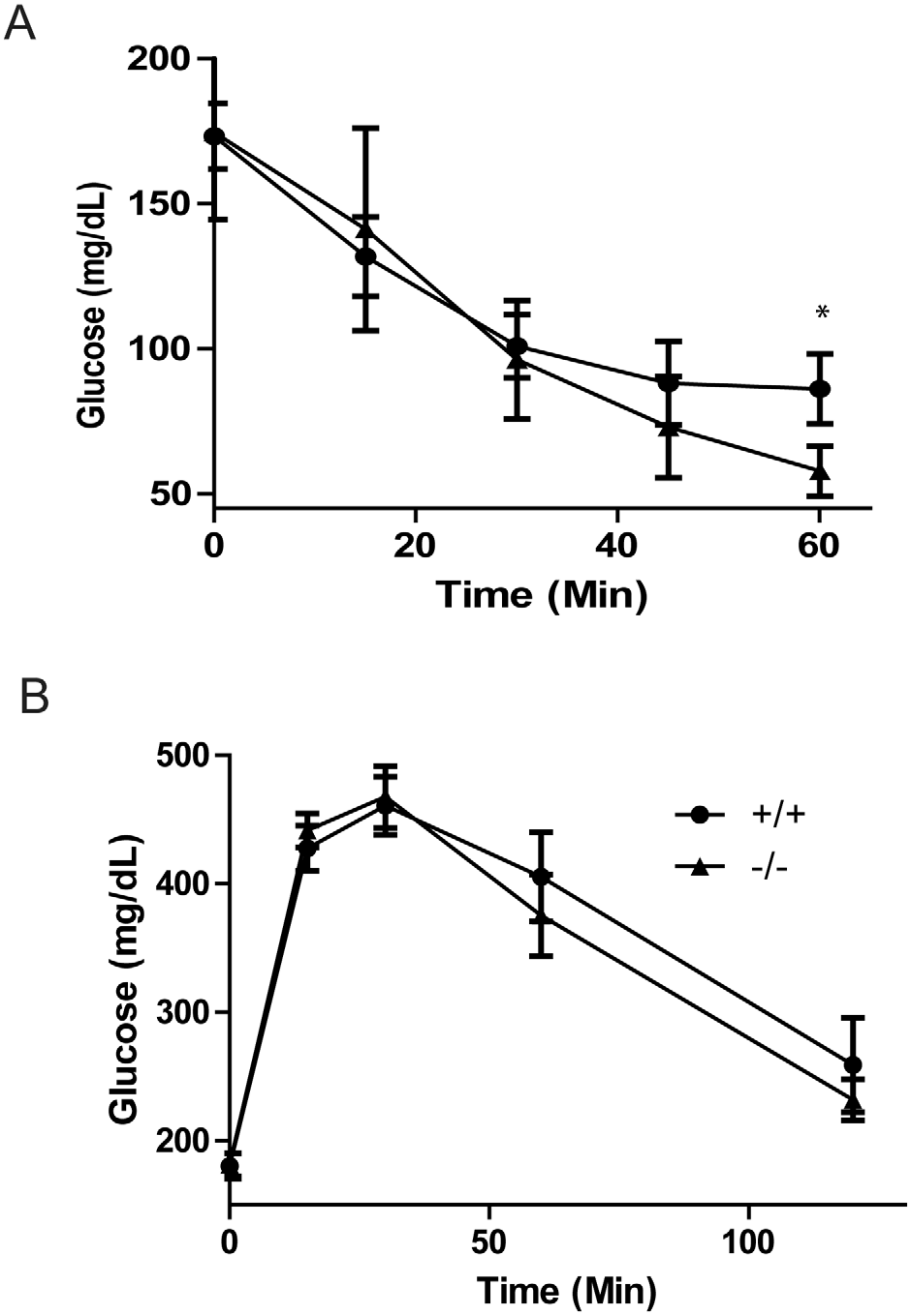
PTPRT KO mice demonstrate better insulin regulation than wild type mice. A) Insulin tolerance test of *Ptprt* ^+/+^ (n = 8) and *Ptprt* ^−/−^ (n = 11) mice after high-fat diet (*p<0.05; t-test). B) Glucose tolerance test of *Ptprt* ^+/+^ (n = 8) and *Ptprt* ^−/−^ (n = 11) mice after high-fat diet.

We then further measured peripheral insulin resistance in mice fed the high-fat diet via an insulin tolerance test. Consistent with the HOMA-IR calculation, *Ptprt* ^−/−^ mice had a lower glucose value in response to an insulin bolus after 60 minutes (Figure 5A). However, *Ptprt* ^−/−^ and *Ptprt* ^+/+^ mice did not have different glucose clearance in response to a glucose tolerance test (Figure 5B). Taken together, our data suggest that loss of PTPRT function attenuates the development of peripheral insulin resistance after a high-fat diet.

### Metabolic Differences between *Ptprt* ^+/+^ and *Ptprt* ^−/−^ Littermates

**Figure 6:**
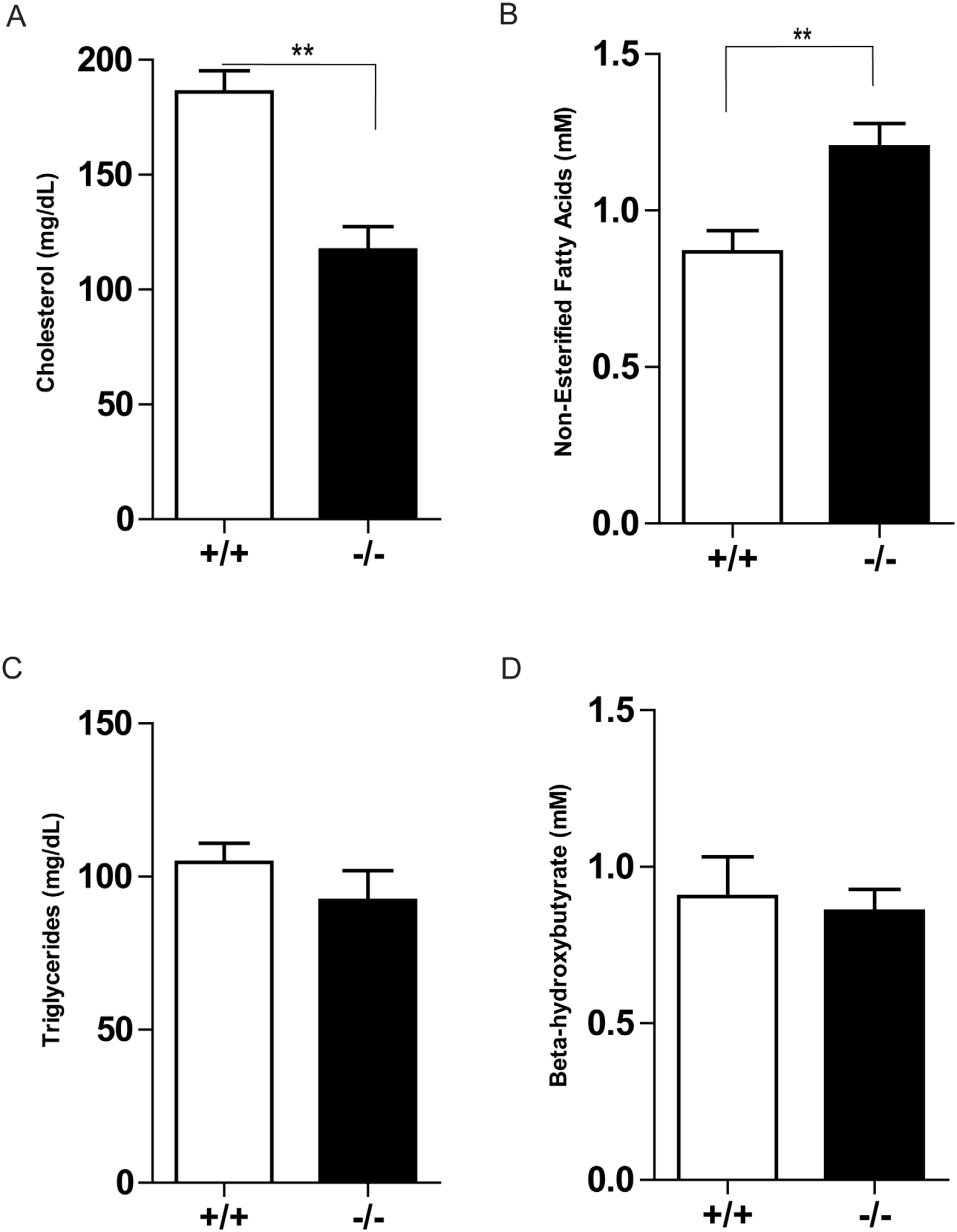
PTPRT KO mice have different blood chemistry values after high-fat diet. Fasted plasma concentrations of cholesterol (A), non-esterified fatty acids (B), triglycerides (C) and beta-hydroxybutyrate (D) of *Ptprt* ^+/+^ (n = 8) and *Ptprt* ^−/−^ (n = 11) mice after high-fat diet (**p<0.01; t-test).

Given the deviation seen between *Ptprt* ^+/+^ and *Ptprt* ^−/−^ mice as it relates to glucose and insulin metabolism, we decided to interrogate their blood plasma for differences in other nutrients. These blood metabolites will shed additional light onto the metabolic disturbances that *Ptprt* ^+/+^ mice are experiencing. Interestingly, *Ptprt* ^−/−^ mice had lower cholesterol and higher free-fatty acids than *Ptprt* ^+/+^ mice, but we did not observe an increase in triglycerides or β-hydroxybutyrate in these mice (Figure 6). Once fatty acids are oxidized, the acetyl CoA produced is used to generate ketone bodies such as β-hydroxybutyrate. As such, PTPRT may also regulate the utilization and storage of dietary fats.

### Phospho-STAT3 Increased in the Hypothalamus of *Ptprt* ^−/−^ Mice

**Figure 7:**
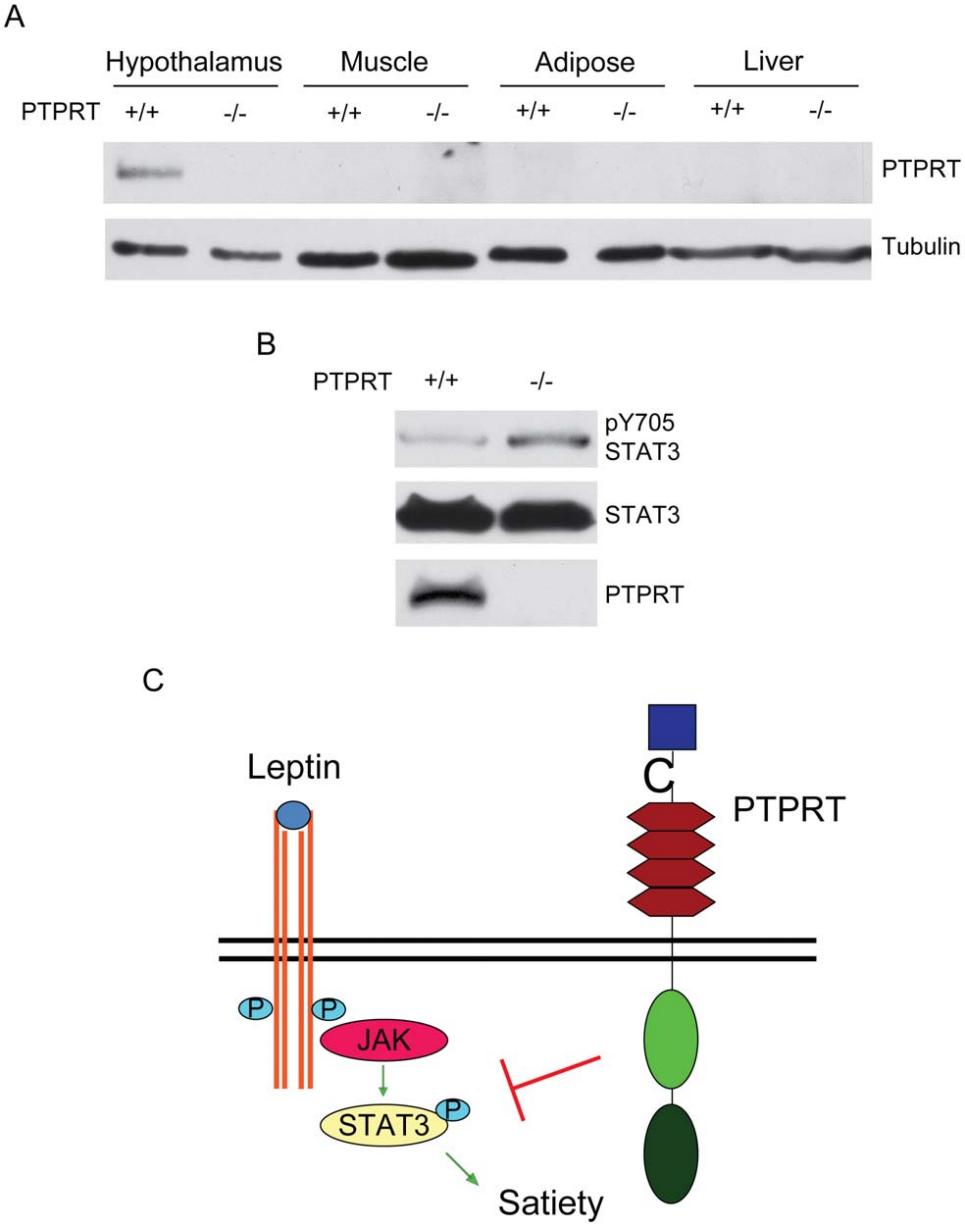
PTPRT regulates STAT3 phosphorylation in mouse hypothalamus. A) Tissue lysates from *Ptprt* ^+/+^ and *Ptprt* ^−/−^ mice were blotted with the indicated antibodies. B) Hypothalamic lysates from *Ptprt* ^+/+^ and *Ptprt* ^+/−^ mice were blotted with the indicated antibodies. C) Proposed model for the effect of PTPRT on food intake.

To elucidate the molecular mechanisms by which *Ptprt* ^−/−^ mice resist high-fat diet-induced obesity, we assessed PTPRT expression in tissues implicated in metabolic regulation. Consistent with a previous report that PTPRT is expressed in the hypothalamus [29], we detected PTPRT protein in the hypothalamus using Western blot analyses (Figure 7A). However, it is not expressed in the liver, adipose or muscle. Since STAT3 is a substrate of PTPRT, we reasoned that phospho-STAT3 levels may be increased in the hypothalamus of *Ptprt* ^−/−^ mice. As expected, we found that pY705 STAT3 is up-regulated in hypothalamus of *Ptprt* ^−/−^ mice compared to *Ptprt* ^+/+^ mice (Figure 7B), suggesting that PTPRT modulates food intake by affecting phospho-STAT3 levels in the hypothalamus (Figure 7C).

## Discussion

Our study reveals that PTPRT regulates metabolism and body weight. Our data suggest that PTPRT could be a drug target for obesity, because *Ptprt* ^−/−^ mice resist many key effects of a high-fat diet, including increased body mass, hyperglycemia, hypercholesterolemia, insulin resistance and increased adiposity. Consistent with this notion, several recent human genetic studies linked obesity to chromosome 20q12–13 [30]–[32], the genomic region in which PTPRT is located.

The decreased food intake in *Ptprt* ^−/−^ mice suggests a behavioral mechanism as to why they weigh less than their *Ptprt* ^+/+^ littermates. Food intake is primarily decreased by leptin signaling pathway [11], [21], [22] and increased by neuropeptide Y [14], [15], [33]. Leptin suppresses food intake by activating STAT3 phosphorylation in the hypothalamus [19], [20]; our previous study shows that PTPRT dephosphorylates STAT3 in colorectal cancers [23]. The decreased food intake in *Ptprt* ^−/−^ mice in the absence of increased circulating leptin levels suggests that *Ptprt* ^−/−^ mice have increased phospho-STAT3 independent of leptin activity in the hypothalamus (Figure 7C). Our data indicate that STAT3 hyper-phosphorylation in the hypothalamus represses food intake in *Ptprt* ^−/−^ mice. As such, we propose that the central nervous system plays a dominant role in the phenotype of *Ptprt* ^−/−^ mice.

*Ptprt* ^−/−^ mice demonstrate decreased peripheral insulin resistance as well as lower levels of blood insulin and glucose. Although a human study shows that PTPRT expression levels in adipose tissue are much higher in insulin-resistant individuals compared to insulin-sensitive individuals [34], we failed to detect PTPRT protein in mouse adipose (Figure 7A). Neither could we detect PTPRT protein in liver or muscle (Figure 7A). Our data indicate that PTPRT does not directly modulate insulin sensitivity in peripheral tissues. Instead, PTPRT may indirectly impact peripheral insulin resistance through affecting the nervous system control of energy homeostasis. It is well documented that increased plasma NPY from autonomic nervous system sources is associated with greater adiposity and increased insulin resistance [35]–[40]. Consistently, our data show increased NPY secretion in *Ptprt* ^+/+^ mice that go on to develop obesity and insulin resistance. As such, the decrease in NPY in *Ptprt* ^−/−^ mice further suggests the role of PTPRT in nervous system regulating obesity and peripheral insulin resistance [41]–[46].

## Materials and Methods

### Animals and Diet

Treatment of experimental mice and related protocols were done in accordance with the Institutional Animal Care and Use Committee at Case Western Reserve University (CWRU). The protocol (Number 2010-0125) was approved by the IACUC Committee at CWRU. Male and female PTPRT heterozygous and homozygous knockout mice in a C57BL/6 background were generated as described previously [24]; referred to as *Ptprt* ^+/−^ and *Ptprt* ^−/−^, respectively. Colonies of these mice were maintained on a normal chow diet (#5010 (4.5% fat by weight, 12.7% fat by calorie), LabDiet St. Louis, MO). Five weeks after birth, male *Ptprt* ^+/+^, *Ptprt* ^+/−^ and *Ptprt* ^−/−^ mice were put on a high-fat diet (#D12331: 33% Hydrogenated Coconut Oil (35% fat by weight; 58% fat by calorie), Research Diets, Inc. New Brunswick, NJ) for 14 weeks. Body composition was analyzed by quantitative magnetic resonance as described previously [26].

### Glucose and Insulin Tolerance Test

Tests were done as described previously [47], [48]. Mice were deprived of food overnight and then injected intraperitoneally with glucose (2 g/Kg) or insulin (0.9 g/Kg). Tail vein blood was sampled for glucose levels at 0, 15, 30 and 60 minutes after insulin or 0, 15, 30, 60 and 120 minutes after glucose using an UltraTouch meter. GTT was performed at the Mouse Metabolic Phenotyping Center of CWRU.

### HOMA-IR

Insulin resistance was estimated for *Ptprt* ^+/+^ and *Ptprt* ^−/−^ mice after high-fat diet using the homeostatic model assessment [28]. Formula = (Insulin (mcU/L)×Glucose (mg/dl)/405).

### Insulin, Neuropeptide Y and Blood Chemistry Measurements

Tests were done as described previously [48]. Mouse blood plasma was isolated using Microtainer plasma separator tubes (BD Biosciences). Insulin was measured using an insulin enzyme-linked immunosorbent assay (Mercodia, Inc., Uppsala, SWE). Neuropeptide Y was measured using neuropeptide Y insulin enzyme-linked immunosorbent assay (EMD Millipore, Billerica, Massachusetts, USA). Mouse plasma was sent to Marshfield Laboratories to assess β-hydroxybutyrate, triglycerides, non-esterified fatty acids and total cholesterol.

### Lipid Absorption

Tests were done as described previously [49]. Mice were fed a diet consisting of butter oil and 5% sucrose polybehenate for three days. Fecal pellets from these mice were collected and analyzed via gas chromatography of fatty acid methyl esters. The ratio of behenic acid to other fatty acids then was used to determine intestinal lipid absorption [27]. The lipid absorption studies were performed at the Cincinnati Mouse Metabolic Phenotyping Center.

### Glucose-stimulated Insulin Secretion Test

Tests were done as described previously [50]. For the glucose-stimulated insulin secretion test, mice were starved overnight and 2 g/kg of glucose was injected intraperitoneally into the mice. Tail vein blood was collected and plasma insulin concentrations were measured at 0 and 30 minutes after glucose injection.

### Indirect Calorimetry

Tests were done as described previously [51]. Indirect calorimetry (IDC) was performed using the Oxymax system (Columbus Instruments’ Comprehensive Lab Animal Monitoring System (CLAMS), Columbus, OH). VO2, VCO2, respiratory quotient (RQ) and heat (energy expenditure – EE) were determined. Energy expenditure was normalized to body mass. IDC was performed on mice either after an overnight fast and water (Fasted) or with ad lib food and water (Fed). The experiments ran for 22 hours on a 12 hour dark cycle (6 pm to 6 am).

### Food Intake

Mice with ad lib access to food and water were placed in a clean cage and the food was weighed. The remaining food after 24 hours was weighed and the average food intake per mouse was calculated [47].

### Western Blot

Tissues were lysed as described previously [52]. Total brain, hypothalamus, hind leg muscle, perigonadal adipose or liver tissue were lysed in RIPA lysis buffer (150 mM NaCl, 10 mM Tris-HCl (pH 7.5), 0.1% SDS, 1% Triton X-100, 1% Deoxycholate, 0.5 M 5 mM EDTA) supplemented with protease (Roche, Penzberg, GER) and phosphatase inhibitors (1 mM NaVO4, 50 mM NaF). Western blots were performed as described previously [24]. Antibodies for pSTAT3^Y705^ and STAT3 were from Cell Signaling (Danvers, MA, USA), tubulin from Sigma-Aldrich (St. Louis, MO, USA) and PTPRT from Biovendor (Asheville, NC, USA).

### Statistical Analysis

Results were assessed using two-tailed unpaired Student’s *t*-test with significance set at *p*<0.05.

## Supporting Information

Figure S1 **PTPRT KO mice demonstrate slightly lower body weight than wild type littermates on normal chow diet.** Eight week-old male mice of *Ptprt* ^+/+^ (n = 13), *Ptprt* ^+/−^ (n = 13) and *Ptprt* ^−/−^ (n = 13) genotypes were maintained on a normal chow diet for 29 weeks. Body weight of the three genotypes was assessed weekly. (*p<0.05; t-test comparing *Ptprt* ^+/+^ and *Ptprt* ^−/−^ genotypes).
(TIF)

Figure S2 **PTPRT KO mice do not have different circulating levels of leptin.** A) Fasting plasma leptin levels of *Ptprt* ^+/+^ and *Ptprt* ^−/−^ mice were assessed before high-fat diet. B) Fasting plasma leptin levels of *Ptprt* ^+/+^ and *Ptprt* ^−/−^ mice were assessed after 14 weeks on a high-fat diet.
(TIF)

Figure S3 **PTPRT KO mice have decreased NPY levels before high-fat diet.** A) Fasting plasma neuropeptide Y levels of *Ptprt* ^+/+^ and *Ptprt* ^−/−^ mice were assessed before high-fat diet (**p<0.01; t-test). B) Fasting plasma neuropeptide Y levels of *Ptprt* ^+/+^ and *Ptprt* ^−/−^ mice were assessed after 14 weeks on a high-fat diet.
(TIF)
