# Supplementary figures and images for: PTPRT Regulates High-Fat Diet-Induced Obesity and Insulin Resistance
Source: PLoS One. 2014 Jun 20;9(6):e100783. doi: 10.1371/journal.pone.0100783 (PMC4065109; doi:10.1371/journal.pone.0100783)

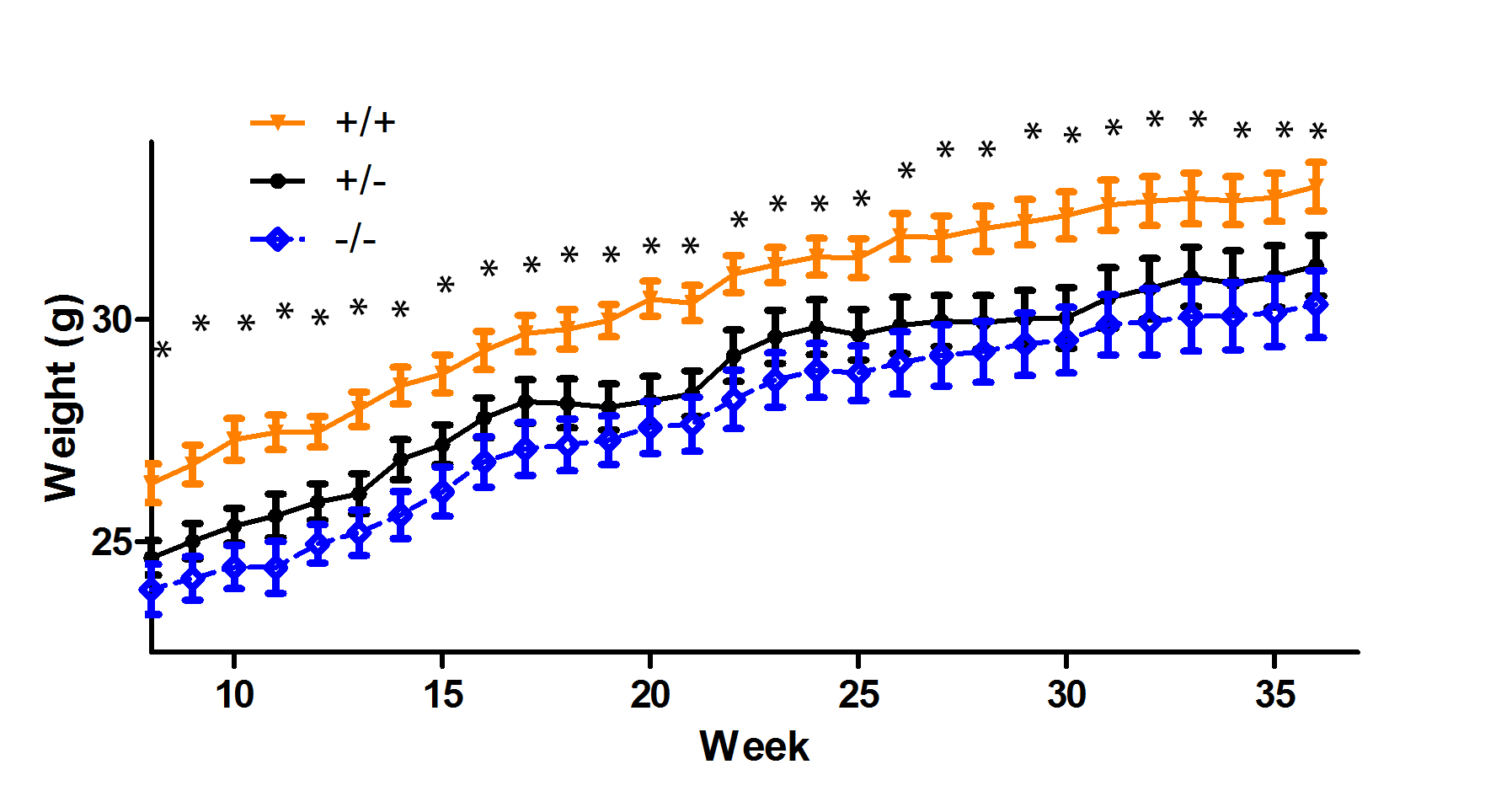

Supplement: Figure S1 — PTPRT KO mice demonstrate slightly lower body weight than wild type littermates on normal chow diet. Eight week-old male mice of Ptprt +/+ (n = 13), Ptprt +/− (n = 13) and Ptprt −/− (n = 13) genotypes were maintained on a normal chow diet for 29 weeks. Body weight of the three genotypes was assessed weekly. (*p<0.05; t-test comparing Ptprt +/+ and Ptprt −/− genotypes). (TIF) [file pone.0100783.s001.tif]

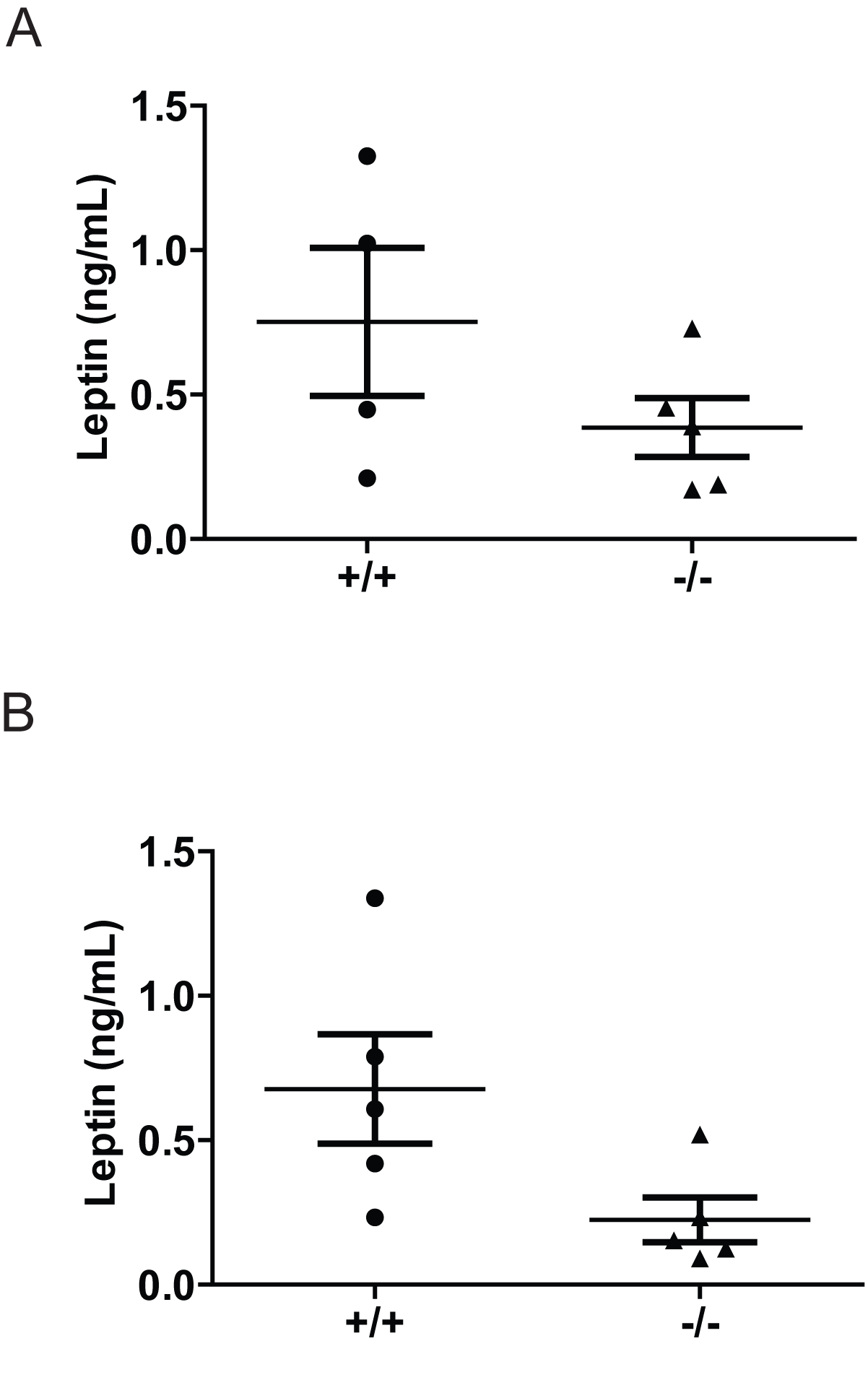

Supplement: Figure S2 — PTPRT KO mice do not have different circulating levels of leptin. A) Fasting plasma leptin levels of Ptprt +/+ and Ptprt −/− mice were assessed before high-fat diet. B) Fasting plasma leptin levels of Ptprt +/+ and Ptprt −/− mice were assessed after 14 weeks on a high-fat diet. (TIF) [file pone.0100783.s002.tif]

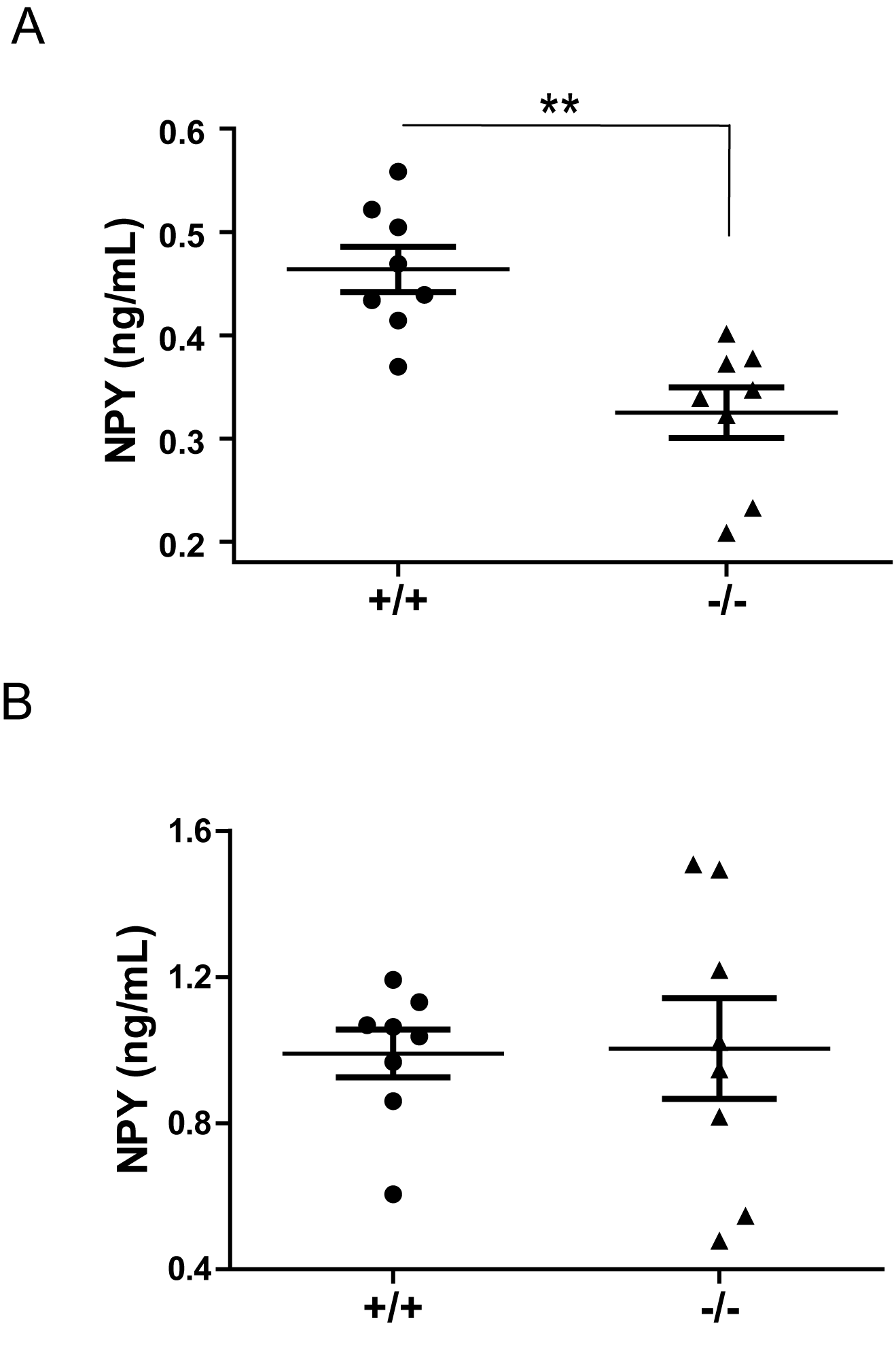

Supplement: Figure S3 — PTPRT KO mice have decreased NPY levels before high-fat diet. A) Fasting plasma neuropeptide Y levels of Ptprt +/+ and Ptprt −/− mice were assessed before high-fat diet (**p<0.01; t-test). B) Fasting plasma neuropeptide Y levels of Ptprt +/+ and Ptprt −/− mice were assessed after 14 weeks on a high-fat diet. (TIF) [file pone.0100783.s003.tif]
